# Hip Morphology–Based Osteoarthritis Risk Prediction Models: Development and External Validation Using Individual Participant Data From the World COACH Consortium

**DOI:** 10.1002/acr.25629

**Published:** 2026-01-14

**Authors:** Myrthe A. van den Berg, Fleur Boel, Michiel M. A. van Buuren, Noortje S. Riedstra, Jinchi Tang, Harbeer Ahedi, Nigel K. Arden, Sita M. A. Bierma‐Zeinstra, Cindy G. Boer, Flavia M. Cicuttini, Timothy F. Cootes, David T. Felson, Willem Paul Gielis, Joshua Heerey, Graeme Jones, Stefan Kluzek, Nancy E. Lane, Claudia Lindner, Joyce B. J. van Meurs, Andrea Mosler, Amanda E. Nelson, Michael C. Nevitt, Edwin H. Oei, Jos Runhaar, Harrie Weinans, Jesse H. Krijthe, Rintje Agricola

**Affiliations:** ^1^ Erasmus MC, University Medical Center Rotterdam Rotterdam The Netherlands; ^2^ University of Tasmania, Menzies Institute for Medical Research, Hobart, Tasmania, and Sydney Orthopaedic Research Institute Sydney New South Wales Australia; ^3^ University of Oxford, Botnar Research Centre Oxford United Kingdom; ^4^ Monash University Melbourne Victoria Australia; ^5^ The University of Manchester Manchester United Kingdom; ^6^ Boston University School of Medicine Boston Massachusetts; ^7^ University Medical Center Utrecht Utrecht The Netherlands; ^8^ La Trobe University Melbourne Victoria Australia; ^9^ University of Tasmania, Menzies Institute for Medical Research Hobart Tasmania Australia; ^10^ University of Oxford, Botnar Research Centre, Oxford, and University of Nottingham Nottingham United Kingdom; ^11^ University of California at Davis Davis; ^12^ University of North Carolina at Chapel Hill Chapel Hill; ^13^ University of California San Francisco; ^14^ University Medical Center Utrecht, Utrecht, and Delft University of Technology Delft The Netherlands; ^15^ Delft University of Technology Delft The Netherlands

## Abstract

**Objective:**

This study aims to develop hip morphology‐based radiographic hip osteoarthritis (RHOA) risk prediction models and investigates the added predictive value of hip morphology measurements and the generalizability to different populations.

**Methods:**

We combined data from nine prospective cohort studies participating in the Worldwide Collaboration on OsteoArthritis prediCtion for the Hip (World COACH) consortium. RHOA grades were harmonized, and incident RHOA was defined as hips without definite RHOA at baseline that developed definite RHOA within four to eight years. Baseline hip morphology was quantified with automatically and uniformly determined lateral center edge angle and alpha angle measurements on anteroposterior radiographs. Discriminative performance of generalized linear mixed model (GLMM) definitions with and without hip morphology measurements was determined with stratified cross‐validation. With leave‐one‐cohort‐out cross‐validation, the generalizability to unseen populations of hip morphology–based GLMMs and random forest (RF) models was evaluated.

**Results:**

From the included 35,984 hips without definite RHOA at baseline, 4.7% developed incident RHOA within four to eight years. The GLMM with cohort‐specific intercept, considering baseline demographics, RHOA grade, and hip morphology measurements, showed a mean area under the receiver operating characteristic curve (AUC) of 0.80 (±0.01) in stratified cross‐validation. Using a marginal intercept decreased performance by 0.1 in AUC. Similar results were found for a GLMM without hip morphology measurements. Leave‐one‐cohort‐out cross‐validation showed comparable discrimination (AUC between 0.56–0.88) and calibration performance for hip morphology‐based GLMMs and RF models.

**Conclusion:**

In hips free of definite RHOA, our AUCs for the incident RHOA models showed good predictive performance in similar populations. However, the added predictive value of the morphology measurements was small, and model performance was heterogeneous in leave‐one‐cohort‐out cross‐validation.

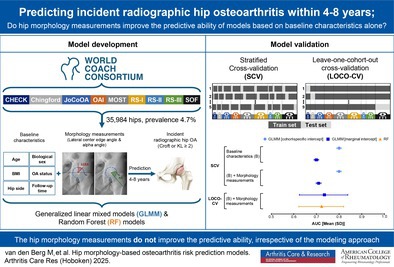

## Graphical abstract

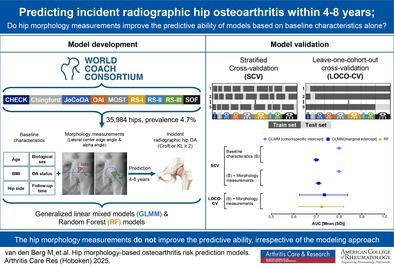

## INTRODUCTION

Despite the growing burden of hip osteoarthritis (HOA) on society and health care systems, prevention and treatment approaches are still only slowly emerging.^1^ Early identification of HOA is important for improving clinical decision‐making and advancing our understanding of disease development and potential prevention and treatment options.^2^ By gaining insights into risk factors and thereby identifying individuals with a high risk of developing HOA, more specific management strategies could be designed for specific subgroups.

**SIGNIFICANCE & INNOVATIONS**

- Our work on hip morphology–based osteoarthritis risk prediction modeling uses an individual participant data meta‐analysis to overcome the limitations of small sample sizes in single‐cohort designs in current epidemiologic research focused on the role of single‐type (eg, cam or pincer) hip morphology on radiographic hip osteoarthritis risk.
- Combining data from 35,984 hips from all available prospective cohort studies with consecutive hip imaging data worldwide (via World COACH), our work uses advanced statistical models to evaluate the importance of continuous hip morphology measures on radiographic hip osteoarthritis (RHOA) risk.
- Although hip morphology was expected to enhance identification of individuals at risk for RHOA, based on its previously established association with RHOA development in isolation, our findings showed that these measures did not improve risk prediction beyond baseline demographics and RHOA grades, highlighting both the complexity of RHOA incidence and challenges in modeling across heterogeneous cohorts.
- Our research demonstrates the importance of large‐scale meta‐analyses for risk modeling and highlights the significance of properly validating risk models in various populations for future clinical application.

Previous research has established that hip morphology plays a key role in the development of radiographic HOA (RHOA).^3^, ^4^ Hip morphology–related conditions that have been shown to increase the risk of developing RHOA include acetabular dysplasia, pincer morphology, and cam morphology. In research settings, these hip morphologies are assessed using cutoff values of continuous morphologic measurements.^5^ However, it is currently recommended to avoid these cutoff values and use the continuous measurements when possible.^6^, ^7^

Current RHOA risk prediction models based on morphology are limited in the level of flexibility and generalizability to unseen data. This problem is caused by the relatively small size of the available datasets and varying study designs.^8^ Therefore, most studies have focused on the risk posed by one type of hip morphology determined by conventional statistical methods.

A larger dataset would allow the investigation of linear and nonlinear interactions between RHOA risk and multiple continuous morphology measurements with flexible statistical and machine learning models. A promising solution to increase the sample size is to combine individual participant data (IPD) of various studies while taking differences between studies into account. These differences could also provide insight into the generalizability of the developed prediction models during internal and external model validation. This approach is expected to increase our understanding of the effect of identified hip morphologies as risk factors on the development of RHOA and could result in more robust prediction models.^9^, ^10^ Additionally, this approach could show if automatic and interpretable assessment of hip morphology on radiographs could enable scalable risk identification and facilitate implementation in everyday clinical settings.

This study aimed to develop a hip morphology–based RHOA risk prediction model by investigating whether including hip morphology measurements could improve the ability of a model to distinguish people with and without RHOA risk within four to eight years. With the use of a large multicohort dataset, we tested the generalizability of our results in both similar and unseen populations.

## MATERIAL AND METHODS

### Study design and participants

This study was designed and reported according to the transparent reporting of multivariable prediction models developed or validated using clustered data (TRIPOD‐Cluster) checklist.^11^ Study participants were selected from the Worldwide Collaboration on OsteoArthritis prediCtion for the Hip (World COACH) consortium. This consortium combines data from prospective cohort studies worldwide with sequential pelvic or hip imaging available. Details of this consortium are published elsewhere.^12^ The current study first considered cohorts with standardized anteroposterior (AP) pelvic, long‐limb, and/or hip radiographs taken and graded for RHOA at baseline and four to eight years of follow‐up. This resulted in the initial inclusion of 38,594 participants from nine cohorts (ie, Cohort Hip and Cohort Knee [CHECK], the Chingford Study, The Johnston County Project [JoCoOA], Multicenter Osteoarthritis Study [MOST], OsteoArthritis Initiative [OAI], Rotterdam Study [RS‐I, RS‐II, RS‐III], and the Study of Osteoporotic Fractures [SOF]).

Additional inclusion criteria were applied to IPD at the hip level. Baseline AP pelvic radiographs were required to have sufficient quality to determine pelvic landmark points for automatic hip morphology assessment. Hips only shown in AP hip (rather than pelvic) radiographs were excluded because a horizontal reference line, needed for adjustment for pelvic tilt, requires both hips in view. Additionally, hips must have been graded for RHOA grade at baseline and follow‐up visit by the original cohort. All hips with the outcomes (ie, definite RHOA or a total hip replacement [THR] at baseline) were also excluded. In total, 35,984 hips (47%, 18,854 participants) with complete data were included in this study.

### Prediction model definition

#### Outcome definition and considered risk factors

RHOA grades were obtained from the original cohort data, all of which used either the Kellgren and Lawrence (KL) or modified Croft grading systems.^13^, ^14^ These scores were harmonized to a three‐grade system: free of RHOA (KL or Croft = 0), doubtful RHOA (KL or Croft = 1), and definite RHOA (KL or Croft ≥2 or THR). Incident RHOA was defined as hips with no definite RHOA at baseline that developed definite RHOA or had a THR within four to eight years. Our baseline population, therefore, included hips with no signs of RHOA (RHOA grade = 0) or doubtful RHOA (RHOA grade = 1).

To assess hip morphology as a risk factor, the lateral center edge angle (LCEA) and alpha angle (AA) were determined uniformly on the baseline radiographs of all included cohorts. Using the BoneFinder software (www.bone-finder.com; The University of Manchester, UK), the outline of the proximal femur and acetabulum was automatically annotated with a custom‐made search model.^15^, ^16^ Subsequently, this point‐set facilitated the automated LCEA and AA measurements through a Python script. Details regarding the reliability and validity of this method have been published previously.^17^ The intermethod intraclass correlation coefficients (ICCs) with 95% confidence interval (CI) between manual and automated measurements were 0.89 (95% CI 0.78–0.94) for the LCEA and 0.46 (95% CI 0.12–0.70) for the AA, similar to the found interobserver ICCs.^16^

**Figure 1:**
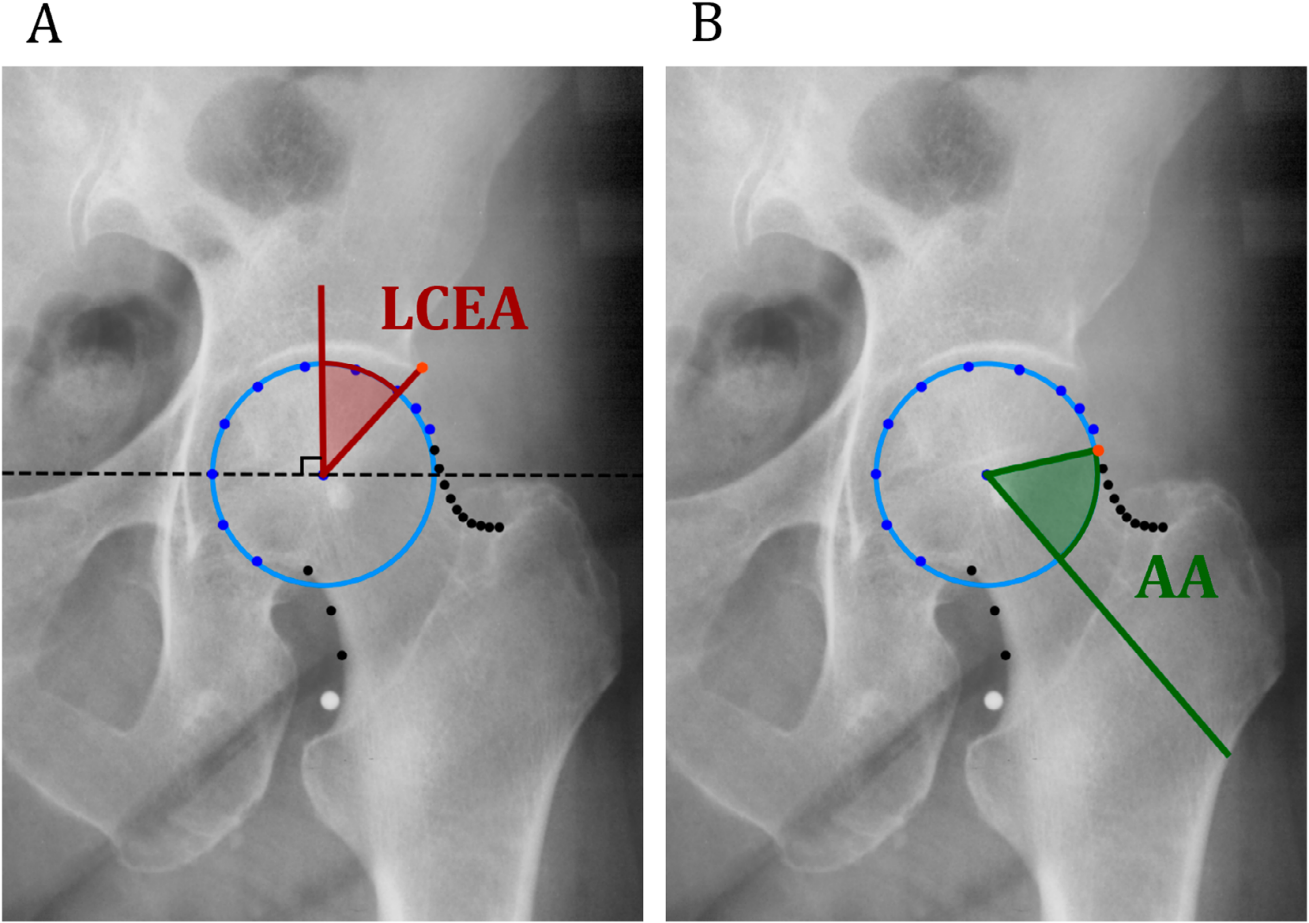
Anteroposterior pelvic radiographs focused on the left hip showing the automatically measured morphology measurements. These methods rely on automated landmark points. To determine the center of the femoral head, a best‐fitting circle (light blue) is outlined around the femoral head based on a subset of femoral head landmark points (dark blue). (A) LCEA (in red): The angle is calculated between a line drawn vertically through the center of the femoral head, perpendicular to the horizontal reference line (black, dashed), and a line originating from the center of the femoral head to the most lateral part of the acetabulum (orange landmark). (B) AA (in green): The angle is calculated between a line through the femoral head center and the point where the femoral head or femoral neck is first outside of the best‐fitting circle (orange landmark) and the femoral neck axis. AA, alpha angle; LCEA, lateral center edge angle.

The LCEA measurement is depicted in Figure 1A. This measurement quantifies the coverage of the femoral head by the acetabulum and is corrected for pelvic tilt. Both under coverage by the acetabulum (smaller LCEA) and over coverage by the acetabulum (larger LCEA) could be potential risk factors for incident RHOA.^3^, ^18^, ^19^, ^20^, ^21^, ^22^, ^23^, ^24^, ^25^ Therefore, we expected a nonlinear relationship between the continuous LCEA measurement and incident RHOA risk.

The AA measurement is depicted in Figure 1B. This measurement describes the degree of sphericity of the lateral femoral head‐neck junction and is used to detect the presence and quantify the degree of cam morphology. A linear relationship between the continuous AA and RHOA is expected, such that the higher the AA (lower sphericity), the higher the risk of incident RHOA.^3^, ^22^, ^23^, ^26^, ^27^

Four different models were defined. Model 1 included demographic information, hip side, and follow‐up time. The demographic information included baseline age, body mass index, and sex assigned at birth, as they are previously proposed risk factors for incident RHOA.^28^ Hip side (left or right hip) and follow‐up time in years were included to account for our hip‐level modeling approach and adjusted for the different follow‐up times of the different cohorts, potentially imposing differences in RHOA risk. In model 2, baseline RHOA grade was added to model 1 to adjust for the expected risk difference for hips with no (RHOA grade 0) and doubtful (RHOA grade 1) RHOA. This risk difference is expected, as hips having an RHOA grade of 1 already show early radiographic changes that can lead to RHOA. After subsequently adding the LCEA and AA measurements in model 3, we considered RHOA grading approach (KL or Croft), cohort location (United States or Europe), and cohort type (open or closed population) as cohort‐describing variables in model 4.

#### Risk prediction modeling approaches

The dataset used is a multilevel dataset, meaning that the data of participants is clustered within cohorts with different cohort‐specific characteristics. In this IPD meta‐analysis, we considered the correlation among hips from the same cohort in our modeling approaches. One of the conventional approaches to handling multilevel data in risk prediction is generalized linear mixed‐effects models (GLMM) for a binary outcome.^29^ For the present study, different GLMMs were built with a binomial distribution and a logit link function. We defined a nested random intercept to account for the correlation of measurements in hips within a participant within a cohort. Ultimately, the estimated marginal model intercept was shifted with a combination of a participant‐specific and cohort‐specific offset value for the participants the model was built on. When new data from the same cohort were used in model testing, the estimated intercept was adjusted with this cohort‐specific adjustment. For participants from new cohorts, the estimated marginal model intercept was used. We considered all risk factors to have a common effect across cohorts and to have no interactions with each other.

As an alternative approach, to step away from the assumptions made in a GLMM model structure, we also built random forest (RF) models that considered cohort variability by including the cohort label as a risk factor. An RF model combines the output of multiple decision trees, making it a nonlinear modeling approach that considers all possible interactions between risk factors. It can also model a nonlinear relationship between the risk factors and incident RHOA.^30^ For individuals from new cohorts, these variables indicating cohort participation would all be set to 0. After initial experiments, hyperparameters on the RF models were set to a maximum depth of 10, number of estimators of 200, and a minimum of eight samples per leaf.

### Statistical analysis

Differences among baseline characteristics of the included and excluded hips and between cohorts were investigated with descriptive statistics. The continuous variables were standardized based on the mean and SD determined on the full dataset before fitting the models. Outcomes were reported based on the original unit scale. Two primary analyses were performed to (1) investigate the associations between the considered risk factors and incident RHOA and to determine the discriminative performance of multiple GLMM definitions; and (2) validate the model performance of both GLMMs and RF models on unseen datasets.

#### (1) Associations between risk factors and incident RHOA and determining the discriminative performance of GLMM definitions

We first compared the adjusted odds ratios (aOR) with 95% CI of the risk factors in four different GLMM specifications. All GLMMs were built with the lme4 package in R version 4.1.1.^31^ The assumed nonlinear effect of the LCEA measurement was modeled with natural cubic splines with two degrees of freedom. Model performance was assessed using stratified five‐fold cross‐validation, in which the relative contribution to the dataset of a cohort and the percentage of hips at risk were kept constant. Therefore, information for cohort‐specific adjustment was available. The discriminative performance of models with or without a cohort‐specific intercept was compared. The area under the receiver operating characteristic curve (AUC) was reported as the mean and SD of the measurement on the five resulting test sets. The AUC classifies the predictive performance as fail (0.5 ≤ AUC < 0.6), poor (0.6 ≤ AUC < 0.7), fair (0.7 ≤ AUC < 0.8), good (0.8 ≤ AUC < 0.9), or excellent (0.9 ≤ AUC).^32^

#### (2) Validation of GLMM and RF models on unseen datasets

To assess the discriminative and calibration performance of both the GLMMs and RF models including the hip morphology measurements to unseen populations, leave‐one‐cohort‐out cross‐validation was used. Each time, a model was built on a dataset of eight cohorts and evaluated on the left‐out cohort. The GLMMs were built with the lme4 package in R version 4.1.1.^31^ The RF models were built with the RandomForestClassifier function in Scikit‐learn version 1.2.0, within Python version 3.9.^33^ The discriminative performance was evaluated by the mean AUC value and 95% CI in the nine left‐out cohorts. Additionally, the calibration statistics of the GLMMs and RF models were compared in each left‐out cohort.

### Data sharing statement

Data are available upon reasonable request. Data may be obtained from a third party and are not publicly available. We encourage the use of data by third parties, although this is subject to approval by the steering committees of the World COACH consortium and the participating cohorts, as well as to legal boundaries regarding data ownership. A standardized data request form is available for which will be reviewed uniformly in order to consistently handle World COACH data requests.

### Patient and public involvement

The World COACH consortium encourages the public to share ideas, stay up to date on our findings, and ask questions through the website (www.worldcoachconsortium.com). Additionally, we help organize annual conferences for patients with OA in the Netherlands (Artrose Gezond, https://artrosegezond.nl/). Patients and public were involved in the design, conduct, reporting, and/or dissemination plans of this research. OA representatives were also involved in setting up the consortium goals and research questions.

### Ethical approval

This study involves human participants but was excepted from ethical approval (Erasmus MC Medical Ethics Review Committee) as it uses previously collected observational data for which the participants had originally given informed consent, and all cohort studies included in this consortium already had ethics approval from their respective committees. Participants gave informed consent to participate in the study before taking part.

## RESULTS

### Participants

**Figure 2:**
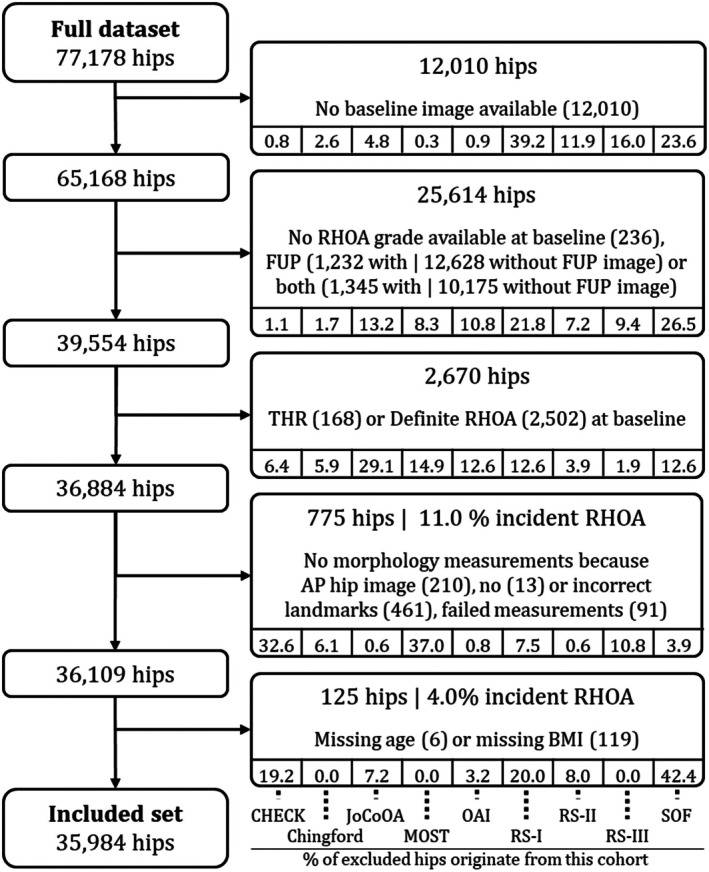
Flow of hips from the fully pooled dataset of the nine cohorts to the final included set. AP, anteroposterior; BMI, body mass index; CHECK, Cohort Hip and Cohort Knee; Chingford, Chingford Study; FUP, follow‐up visit; JoCoOA, Johnston County Project; MOST, Multicenter Osteoarthritis Study; OAI, OsteoArthritis Initiative; RHOA, Radiographic hip osteoarthritis; RS‐I, Rotterdam Study‐I; RS‐III, Rotterdam Study‐III; SOF, Study of Osteoporotic Fractures; Study‐II RS‐II, Rotterdam; THR, total hip replacement.

A detailed overview of the flow of inclusion and exclusion of hips while pooling the data of the nine cohorts to the final included set is shown in Figure 2. The largest proportion (62%) of hips were excluded because these hips were not graded for RHOA at baseline by the cohort or had no radiograph and/or RHOA grade available at follow‐up. Descriptive statistics of the pooled included and excluded dataset, stratified by cohort, are given in Table 1. The excluded population was older than the included population (67.5 ± 10.6 years compared to 63.5 ± 8.5 years). The other descriptive characteristics were similar.

**Table 1:** Descriptive statistics of the included cohorts, the pooled included set, and pooled excluded set*

	CHECK	Chingford	JoCoOA	MOST	OAI	RS‐I	RS‐II	RS‐III	SOF	Included set	Excluded set^a^
Participants											
Excluded participants, n	361	408	2,212	1,310	1,494	5,260	1,671	2,207	4,812		19,735
Included participants, n	641	595	1,772	1,716	3,302	2,860	1,341	1,732	4,895	18,854	
Sample size											
Hips, n	1,189	1,060	3,209	3,197	6,372	5,542	2,624	3,408	9,383	35,984	41,194
Right hips, %	49.9	47.0	51.6	50.4	49.9	49.7	49.6	50.1	49.6	49.9	50.1
Baseline factors											
Age at baseline, mean ± SD, y	55.8 ± 5.22	53.5 ± 5.71	60.1 ± 8.68	61.7 ± 7.72	60.7 ± 9.03	65.3 ± 6.52	63.2 ± 6.56	56.3 ± 5.01	70.6 ± 4.50	63.4 ± 8.47	67.5 ± 10.58
Male hips, n (%)	210 (17.7)	0 (0.0)	1,232 (38.4)	1,096 (34.3)	2,704 (42.4)	2,412 (43.5)	1,159 (44.2)	1,507 (44.2)	0 (0.0)	10,320 (28.7)	11,750 (28.5)
BMI at baseline, mean ± SD, kg/m^2^	26.1 ± 4.00	25.4 ± 3.99	29.5 ± 5.88	29.5 ± 4.85	28.2 ± 4.57	26.3 ± 3.47	27.1 ± 3.90	27.5 ± 4.13	26.5 ± 4.36	27.4 ± 4.57	27.7 ± 5.29
Hips with RHOA grade 1, n (%)	293 (24.6)	71 (6.7)	2,445 (76.2)	1,193 (37.3)	809 (12.7)	2.203 (39.8)	372 (14.2)	322 (9.4)	4,583 (48.8)	12,291 (34.2)	5,784 (27.7)
AA, mean ± SD, degrees	46.4 ± 9.92	51.3 ± 12.63	44.7 ± 8.64	47.1 ± 9.64	48.1 ± 11.45	46.4 ± 10.13	45.5 ± 9.52	50.8 ± 12.84	45.1 ± 9.57	46.7 ± 10.57	47.7 ± 11.75
LCEA, mean ± SD, degrees	34.7 ± 5.59	36.1 ± 6.04	36.3 ± 5.86	33.4 ± 6.05	35.3 ± 5.64	36.6 ± 5.81	35.5 ± 5.68	34.7 ± 5.62	38.2 ± 5.89	36.1 ± 5.99	36.7 ± 6.35
Cohort‐level covariates											
Follow‐up time, y	8	7	6	5	4	8	4	6	8	4–8	4–8
RHOA grading system	KL	KL	KL	KL	Croft	KL	KL	KL	Croft	KL, Croft	KL, Croft
Location of cohort	Europe	Europe	United States	United States	United States	Europe	Europe	Europe	United States	Europe, United States	Europe, United States
Included population type	Closed	Open	Open	Closed	Closed	Open	Open	Open	Open	Open, closed	Open, closed
Outcome of interest											
Hips with incident RHOA, n (%)	240 (20.2)	110 (10.4)	386 (12.0)	133 (4.2)	95 (1.5)	173 (3.1)	44 (1.7)	146 (4.3)	363 (3.9)	1,690 (4.7)	94 (10.4)

* AA, alpha angle; BMI, body mass index; CHECK, Cohort Hip and Cohort Knee; Chingford, Chingford Study; JoCoOA, Johnston County; KL, Kellgren and Lawrence grade; LCEA, lateral center edge angle; MOST, Multicenter Osteoarthritis Study; OAI, OsteoArthritis Initiative; RHOA, radiographic hip osteoarthritis; RS‐I, Rotterdam Study‐I; RS‐II, Rotterdam Study‐II; RS‐III, Rotterdam Study‐III; SOF, Study of Osteoporotic Fractures.

^a^ Sample sizes vary for the descriptive statistics of the excluded set as they are based on only the available data for this variable.

In our included set of 35,984 hips free of definite RHOA at baseline, 1,690 hips developed incident RHOA within four to eight years. Whereas the pooled prevalence of incident RHOA was 4.7%, the prevalence among the cohorts ranged from 1.5% for OAI to 20.2% for CHECK. Additionally, the prevalence of doubtful RHOA ranged from 9% in RS‐III to 76% in JoCoOA at baseline. Other differences between the cohort populations at baseline were their mean baseline age, ranging from 54 to 71 years, and sex distribution, with Chingford and SOF being female‐only cohorts.

### Associations of risk factors and incident RHOA and discriminative performance of GLMM definitions

**Figure 3:**
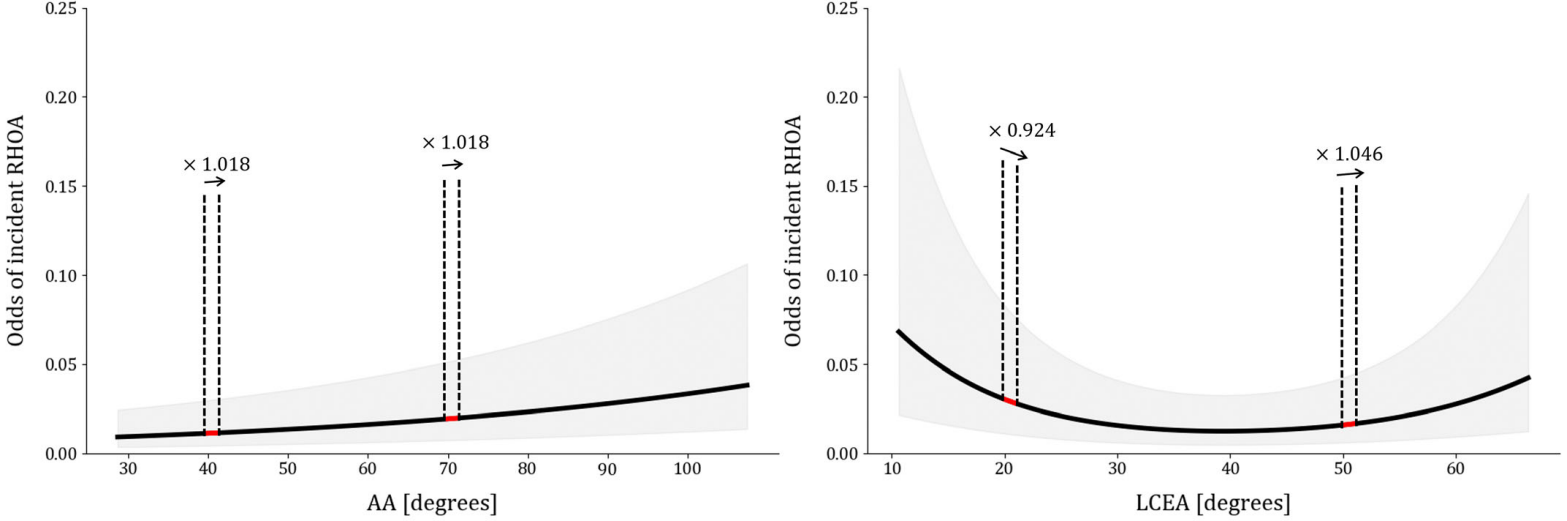
Predictor effect plots of the morphology measurements included in the multivariable risk‐prediction model GLMM3. Odds are given for a female participant after eight years of follow‐up, with the mean values of the continuous factors and the reference values for the categorical factors. The annotated multiplication factors describe the change in odds for an increase of 1° at that location. AA, alpha angle; LCEA, lateral center edge angle; GLMM, generalized linear mixed model; RHOA, radiographic hip osteoarthritis. Color figure can be viewed in the online issue, which is available at http://onlinelibrary.wiley.com/doi/10.1002/acr.25629/abstract.

The aORs and discriminative performance in terms of AUC for our four GLMM definitions are shown in Table 2. Summary statistics on the data used for each train and test dataset combination and model specifications can be found in the Supplementary Material. For the LCEA, the two values of the aOR represent the exponent of the first and second components of the natural cubic spline transformation, respectively. To interpret the effect of 1° of change in the LCEA value, the predictor effect plot for GLMM3 is shown in Figure 3. An aOR of 1.018 for the AA can be interpreted as that, for every 1° increase in the AA value, the odds of incident RHOA increase by 1.8%. Similarly, having an LCEA of 20° compared to 21°, the odds of incident RHOA decrease by 8% (aOR = 0.924).

**Table 2:** Risk factor contribution in terms of aORs of four different multivariate GLMM definitions on the pooled included dataset and their discriminative performance based on stratified cross‐validation*

	GLMM1	GLMM2	GLMM3	GLMM4
Age, in years	**1.051 (1.041–1.062)**	**1.043 (1.033–1.053)**	**1.043 (1.033–1.054)**	1.011 (0.947–1.079)
Sex at birth				
Female [reference]				
Male	1.118 (0.954–1.310)	0.979 (0.830–1.155)	**0.828 (0.695–0.987)**	0.389 (0.113–1.336)
BMI, in kg/m^2^	1.012 (0.998–1.026)	1.012 (0.998–1.027)	1.009 (0.994–1.024)	1.023 (0.920–1.138)
Hip side				
Left [reference]				
Right	1.101 (0.987–1.229)	**1.149 (1.025–1.290)**	**1.158 (1.031–1.301)**	**1.476 (1.206–1.806)**
Follow‐up time, in years	**1.511 (1.124–2.032)**	1.410 (0.928–2.144)	1.409 (0.942–2.106)	1.212 (0.764–1.923)
RHOA grade				
Free of RHOA [reference]				
Doubtful RHOA		**9.000 (7.602–10.655)**	**8.751 (7.378–10.38)**	**17.820 (11.768–26.985)**
AA, in degrees			**1.018 (1.012–1.024)**	1.018 (0.999–1.038)
LCEA,^a^ in degrees				
First spline component			**0.062 (0.019–0.197)**	0.009 (0.000–0.390)
Second spline component			1.771 (0.856–3.662)	4.931 (0.528–46.029)
Scoring approach				
KL [reference]				
Croft				0.353 (0.074–1.677)
Cohort type				
Open [reference]				
Closed				2.593 (0.775–8.672)
Location				
Europe [reference]				0.483 (0.115–2.029)
United States				
AUC				
With cohort‐specific intercept	0.72 ± 0.01	0.80 ± 0.01	0.80 ± 0.01	0.76 ± 0.02
With marginal intercept	0.56 ± 0.01	0.70 ± 0.01	0.71 ± 0.01	0.75 ± 0.01

* Values are in aOR (95% confidence interval) or mean ± SD. Bold numbers indicate a significant association (*P* < 0.05). AA, alpha angle; aOR, adjusted odds ratio; AUC, area under the receiver operating characteristic curve; BMI, body mass index; GLMM, generalized linear mixed model; KL, Kellgren and Lawrence; LCEA, lateral center edge angle; RHOA, radiographic hip osteoarthritis.

^a^ Variable is modeled with natural cubic splines (*df* = 2), displayed aORs are the exponent of the beta values of the two spline components.

When using the cohort‐specific intercept, adding the baseline RHOA grade (ie, GLMM1 vs GLMM2) improved the discriminative performance of the model from a mean AUC of 0.72 to 0.80 (Table 2), whereas the morphology measurements (GLMM3) did not change this performance. Overall, the discriminative performance was ~0.1 in AUC value higher when using a cohort‐specific intercept to predict the risk of incident RHOA in GLMM1, GLMM2, and GLMM3. When adding the cohort descriptive variables as fixed‐effects in the model, the AUC was reduced to 0.76 using the cohort‐specific intercept but increased to 0.75 with the marginal intercept, compared to the other model definitions. In GLMM4, the cohort‐specific adjustments to the intercept were found to be nonexistent. However, the cohort‐specific variables did not show a statistically significant association with incident RHOA. Adding these variables resulted in different aOR values and larger CIs for the other considered risk factors. Specifically, the aOR for having doubtful RHOA at baseline was 17.8. Therefore, the results for GLMM4 should be interpreted with caution.

### GLMM and RF model validation on unseen datasets

Table 3 shows the results of the leave‐one‐cohort‐out validation for both the GLMMs and RF models. These models included the baseline patient characteristics, RHOA grade, and morphology measurements (as defined in GLMM3). The summary statistics on the data used for each train and test dataset combination, model specifications, calibration curves, and receiver operating characteristic curves can be found in the Supplementary Material. The discriminative performance (AUC) ranged from 0.57 to 0.88 and 0.56 to 0.87 for the GLMMs and RF models, respectively. Validation on the OAI and RS‐II cohorts had the highest AUC values, which were the cohorts with the lowest prevalence of incident RHOA. Validation on Chingford and JoCoOA showed the worst performance. The mean predicted values were mostly higher for the RF model, except for RS‐I and SOF. When analyzing the model calibration, we observed that models overestimated the risk for the same two cohorts. For CHECK, JoCoOA, Chingford, and RS‐III, both models underestimated the risk. For MOST, OAI, and RS‐II, the GLMM underestimated and the RF model overestimated the risk. The RF models seemed better calibrated than the GLMM.

**Table 3:** Discriminative performance and mean predicted probabilities of the GLMM and RF models based on leave‐one‐cohort‐out cross‐validation*

Cohort	Risk of incident RHOA		GLMM		RF	
	OA/No‐OA	Prevalence, %	Mean predicted values, %	AUC (95% CI)	Mean predicted values, %	AUC (95% CI)
CHECK	240/949	20.2	0.01	0.69 (0.65–0.73)	3.24	0.68 (0.64–0.72)
Chingford	110/950	10.4	0.91	0.57 (0.51–0.63)	2.26	0.57 (0.51–0.63)
JoCoOA	386/2,823	12.0	4.53	0.57 (0.54–0.60)	7.02	0.56 (0.53–0.59)
MOST	133/3,064	4.2	2.25	0.80 (0.77–0.83)	5.33	0.80 (0.76–0.83)
OAI	95/6,277	1.5	0.88	0.86 (0.82–0.90)	3.60	0.82 (0.77–0.87)
RS‐I	173/5,369	3.1	9.72	0.78 (0.75–0.80)	5.99	0.76 (0.73–0.79)
RS‐II	44/2,580	1.7	0.97	0.88 (0.83–0.94)	3.01	0.87 (0.80–0.93)
RS‐III	146/3,262	4.3	1.04	0.75 (0.70–0.80)	2.44	0.74 (0.69–0.79)
SOF	363/9,020	3.9	14.61	0.69 (0.66–0.71)	7.16	0.69 (0.67–0.72)

* AUC, area under the receiver operating characteristic curve; CHECK, Cohort Hip and Cohort Knee; Chingford, Chingford Study; CI, confidence interval; GLMM, generalized linear mixed model; JoCoOA, Johnston County Project; MOST, Multicenter Osteoarthritis Study; OAI, OsteoArthritis Initiative; RF, random forest; RHOA, radiographic hip osteoarthritis; RS‐I, Rotterdam Study‐I; RS‐II, Rotterdam Study‐II; RS‐III, RS‐III, Rotterdam Study‐III; SOF, Study of Osteoporotic Fractures.

## DISCUSSION

We thoroughly investigated the effect of two continuous hip morphology measurements on incident RHOA within four to eight years in a large international IPD analysis of nine cohorts and nearly 36,000 hips. During stratified cross‐validation, similar discriminative performance was found for models with and without the LCEA and AA measurements (mean AUC 0.80 ± 0.01). When comparing GLMM and RF modeling strategies and their generalizability to unseen populations, similar performances were found with AUC values ranging between 0.56 and 0.88 for each left‐out cohort. This implies that using a more flexible model definition that includes possible risk factor interactions or nonlinear relationships would not improve the models. The RF models were better calibrated, which implies that the estimated probabilities are more consistent with the observed RHOA risk distribution within the population.

Previous studies examining the role of hip shape in RHOA risk primarily converted the measurements into a binary risk factor using specific thresholds. Considering hip morphology measurements as a continuous risk factor allows for a more data‐driven estimation of the effect. Despite recognizing several hip morphologies as RHOA risk factors, inconsistencies in the literature regarding the thresholds used complicate the interpretation of reported associations.^34^ In agreement with previous reports, we found increased odds of incident RHOA when the morphologies were present.^21^, ^22^, ^23^, ^35^

In contrast, we found comparable AUC values when evaluating the discriminative performance of the models with and without hip morphology measurements (GLMM3 vs GLMM2). This indicates that inclusion of the AA and LCEA did not improve the ability of the model to distinguish between hips that developed or did not develop incident RHOA. Possible explanations are that these geometric measurements alone are not descriptive enough to capture the structural differences between individuals at and not at risk for RHOA development. The low to moderate interrater and intermethod ICCs for the AA might also play a role, potentially reducing the sensitivity of the measurement to identify individuals at risk. Alternatively, abnormal hip morphology may be a true risk factor for early‐onset hip OA, but the high‐risk participants are already excluded from this analytic population.^23^

The largest increase in AUC values was observed when adding the baseline RHOA status to our model. This indicates that doubtful or mild radiographic changes (ie, RHOA grade 1) identified hips at risk for incident RHOA within four to eight years. Our findings are comparable to those in Saberi Hosnijeh et al, in which an increase of 0.11 in AUC on the training set (RS‐I) was found when the imaging variables baseline KL score, thumb OA, hip dysplasia, and cam morphology were added to their basic model; a similar increase in AUC values was found in external validation on CHECK and RS‐II data.^36^ Based on these findings, in combination with our results, baseline RHOA grade was expectedly the largest contributor to this increase in AUC. Additionally, where Saberi Hosnijeh et al showed an aOR of 4.43 (3.14–5.77) for having KL grade 1, we found an aOR of 8.75 (7.38–10.38) for having either KL or Croft grade 1 in GLMM3. Although various opinions exist on considering no versus doubtful RHOA within models for incident RHOA to better understand the specific role of risk factors, including baseline RHOA status in prediction models better identifies hips at risk.

Additionally, differences in performance were found when a cohort‐specific intercept instead of a marginal intercept was used. This implies that the currently considered variables do not explain the heterogeneity in incident RHOA prevalence between the cohorts. When cohort‐level covariates were added to account for variability in inclusion criteria and cohort setup, the marginal and cohort‐specific intercepts were identical. However, because the number of cohorts is always going to be limited, it is inherently difficult to estimate the cohort‐level parameters accurately. We hypothesize that the heterogeneity in incident RHOA prevalence is mainly the result of the differences in cohort setup (ie, follow‐up time and specific inclusion of people at risk of OA), the semiobjective nature of the RHOA scoring systems, and the different scoring approaches used by the cohorts.^37^ Future work in the World COACH consortium aims to include standardized RHOA scoring across cohorts to ensure that observed heterogeneity reflects actual population differences rather than inconsistencies in scoring methods.

The heterogeneity between cohorts contributed to heterogeneous model performance for leave‐one‐cohort‐out validation. Overall, we observed that when the prevalence in the training dataset was higher than the prevalence in the test dataset, discriminative performance improved. As noted by Riley et al, even when the found predictor effects are consistent, variations in characteristics or prevalence across different settings or populations can lead to genuine differences in model calibration due to incorrect estimation of the intercept.^38^ In general, an underestimation of incident RHOA risk was found, except for the two older cohorts RS‐I and SOF. This could indicate that the considered risk factors have a different effect on RHOA risk for this older population than in the younger training population. More efforts in modeling and understanding the underlying factors for incident RHOA prevalence in these different cohorts could therefore improve RHOA risk estimates. Despite our efforts to combine data from multiple cohorts via IPD to consider a more versatile population to better estimate the intercept and risk factors in new populations, we observed spectrum bias.^39^ In addition to creating a larger dataset, the target population should be considered when selecting cohorts to combine, and more risk factors should be added to explain any variability between the cohorts.

The major strength of this study is that it is the first to use data from all available prospective cohort studies on HOA to create risk prediction models for incident RHOA within four to eight years. By combining these IPD, we can include a large and heterogeneous study population. This covers the general population of interest better than individual single‐cohort studies. By uniformly measuring our baseline factors, automatically determining our morphology measurements, and taking the cohort differences into account in our modeling approaches, we could provide a more precise estimation of the performance of our prediction models.^40^ Through evaluation of models via both internal and external validation, we assessed the generalizability of our predictions, which, although highly encouraged, can be challenging for clinical risk prediction models.

The limitations of the study are mainly caused by the differences in setup of the nine included cohorts, such as varying imaging protocols, inclusion criteria, and follow‐up times. Firstly, our defined follow‐up time of four to eight years might have been too short and too broad to determine reliable RHOA risk estimates due to the low prevalence of incident RHOA, and this variability in follow‐up duration could reduce the apparent strength of associations. When considering an extended follow‐up for cohorts with this data available, we see that the prevalence of incident RHOA can double when looking, for example, at 11 years of follow‐up for RS‐I and RS‐II. Secondly, by only defining hip morphology on AP radiographs, we might underestimate its presence and severity, which is better captured in three dimensions. Nevertheless, these automated measurements on two‐dimensional imaging allow for easier screening of large populations. Additionally, as the inclusion criteria were applied at the hip level, one potential unadjusted and unmeasured confounder was the increased risk of incident RHOA in individuals with contralateral RHOA. However, this was not the focus of the study. Moreover, including THR in our definition of incident RHOA might have led to some misclassification, as individuals could have undergone THR for other reasons than hip OA. Nonetheless, this group of people accounts for less than 5% of all incident RHOA cases in our study. Lastly, our outcome definition of incident RHOA was dependent on semiobjective scoring systems with high intra‐ and interrater variability,^41^ and Fan et al have shown that prevalence estimates with the KL and Croft grading systems were statistically different.^1^ Although the defined risk of interest aligns with the current clinical practice, it does limit the interpretability of the results.

Based on the current study, using more automated RHOA grading systems should be encouraged in the future to reduce variability in scoring for multicohort analyses. In addition, automated and uniform RHOA grading methods could reduce the time needed to reliably grade radiographs already collected in epidemiologic research and could therefore increase the sample size. To aid clinical decision‐making and treatment options, multimodal prediction modeling with risk factors from different domains, such as genetics and clinical examination, is recommended.^42^ Although the set of considered risk factors in this study already provided valuable insights into incident RHOA, creating artificial intelligence models that can analyze radiographs and consider many more than only demographic and hip morphology–based risk factors could improve our understanding of HOA development. This might also reduce the present heterogeneity in model performance when applied to new populations.

In conclusion, the hip morphology–based risk prediction models built with multicohort data had good predictive performance for incident RHOA within four to eight years for hips free of definite RHOA at baseline. However, we found relatively high heterogeneity in model performance in leave‐one‐cohort‐out cross‐validation. The added value of hip morphology measurements on the discriminative performance was small compared to a model with baseline patient characteristics and RHOA grade alone. More efforts are recommended to reduce the heterogeneity between cohorts and increase model calibration. Ultimately, risk prediction models built with multicohort data could lead to an improved understanding of HOA and be a first step toward individualized HOA care.

## AUTHOR CONTRIBUTIONS

All authors contributed to at least one of the following manuscript preparation roles: conceptualization AND/OR methodology, software, investigation, formal analysis, data curation, visualization, and validation AND drafting or reviewing/editing the final draft. As corresponding author, Dr Agricola confirms that all authors have provided the final approval of the version to be published, and takes responsibility for the affirmations regarding article submission (eg, not under consideration by another journal), the integrity of the data presented, and the statements regarding compliance with institutional review board/Declaration of Helsinki requirements.

## Supporting information


**Disclosure Form**:

**Appendix S1:** Supplementary Information
