# Supplementary material for: Hip Morphology–Based Osteoarthritis Risk Prediction Models: Development and External Validation Using Individual Participant Data From the World COACH Consortium
Source: Arthritis Care Res (Hoboken). 2026 Jan 14;78(4):489–500. doi: 10.1002/acr.25629 (PMC13034096; doi:10.1002/acr.25629)
Supplement: Supplementary file 2 — Appendix S1: Supplementary Information [file ACR-78-489-s001.docx]

# Supplementary material

## Associations of risk-factors and incident RHOA and discriminative performance of GLMM definitions

Table S1 - Descriptive statistics on datasets used for model building and testing


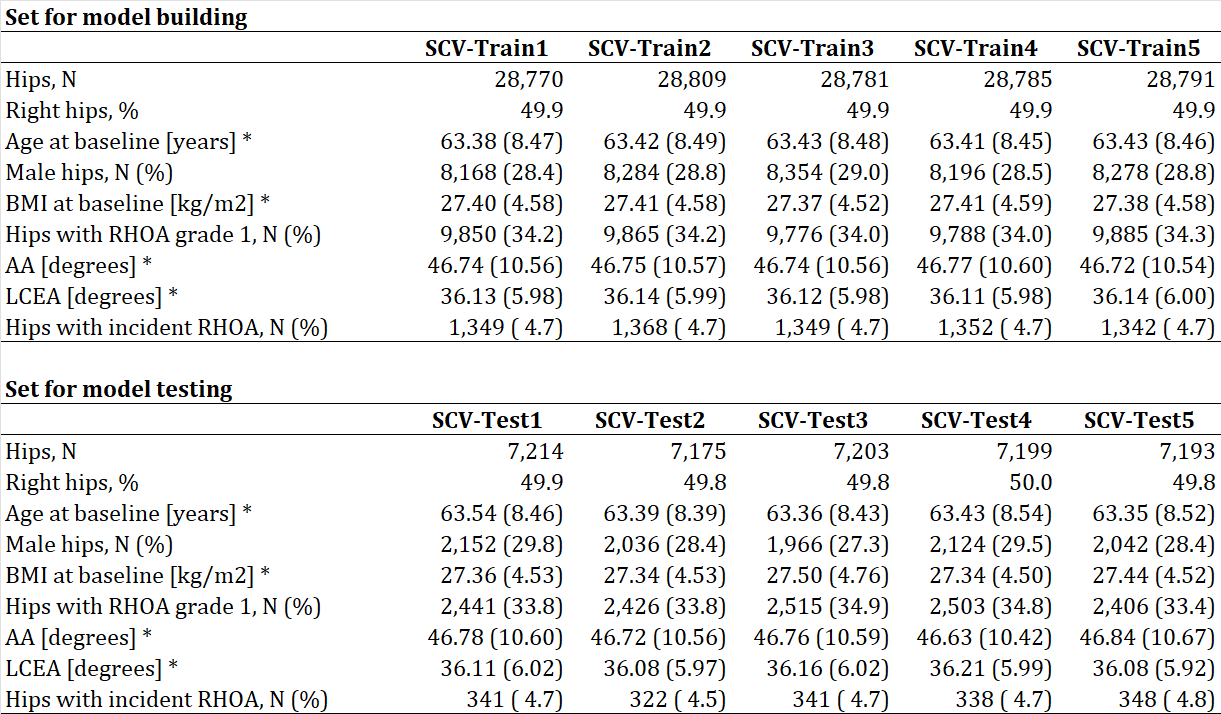


*Stratified cross-validation (SCV), body mass index (BMI), radiographic hip osteoarthritis (RHOA), alpha angle (AA), lateral center edge angle (LCEA)*

Table S2 - Beta values of each of the considered risk-factors of each of the four model definitions


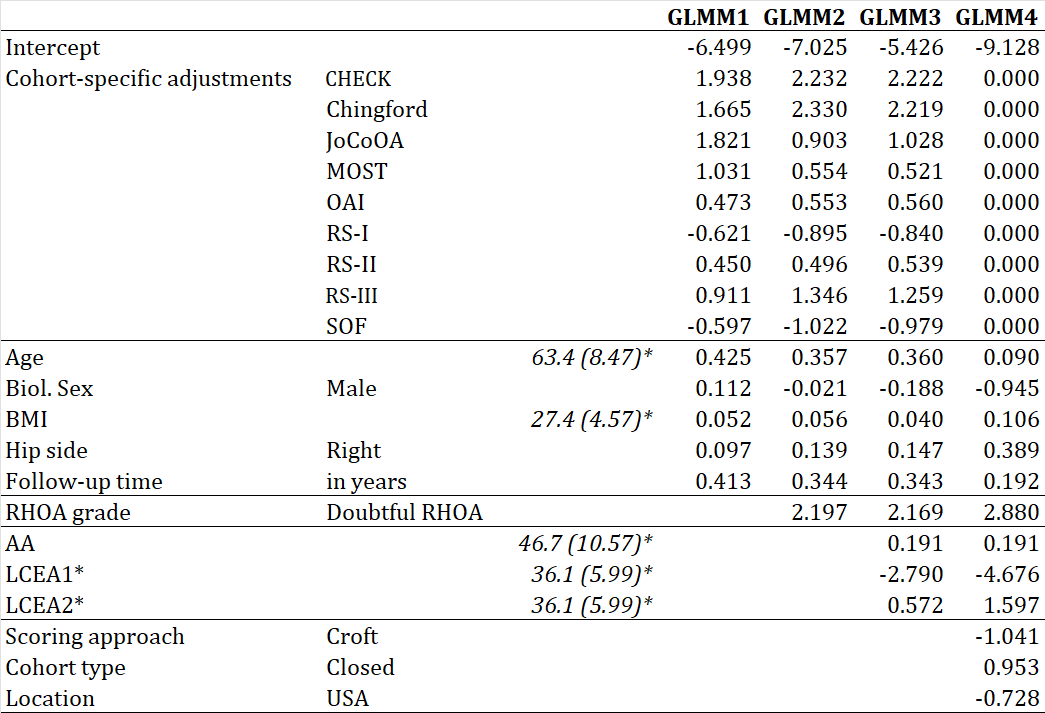


*Generalized Linear Mixed Model (GLMM), Cohort Hip and Cohort Knee (CHECK), Chingford Study (Chingford), Johnston County Project (JoCoOA), Multi-center Osteoarthritis Study (MOST), OsteoArthritis Initiative (OAI), Rotterdam Study-I (RS-I), Rotterdam Study-II (RS-II), Rotterdam Study-III (RS-III), Study of Osteoporotic Fractures (SOF), body mass index (BMI), radiographic hip osteoarthritis (RHOA), alpha angle (AA) lateral center edge angle (LCEA), Kellgren and Lawrence (KL), United States of America (USA)*

**Mean (SD) values used to standardize the input data*

*** variable is modelled with natural cubic splines (df = 2), first and second beta values are given*

CodeBlock S1 – R script for building the four different GLMM models and predicting the risk with GLMM1

| # Load all packages  library(readxl)  library(lme4)  library(dplyr)  library("splines")  library(groupdata2)  set.seed(1)  # Import dataset for analysis & select complete cases  df = read_excel("PATH TO DATASET")  df <- df[complete.cases(df), ]  # Transform data into right format  df$cohort_name = factor(df$cohort_name,  levels=c("CHECK","CHINGFORD","JOCO","MOST","OAI","SOF","RS1", "RS2","RS3"))  df$person_id = factor(df$person_id)  df$age = as.numeric(df$age)  df$bmi = as.numeric(df$bmi)  df$sex <- factor(df$sex)  df$hip_side = factor(df$hip_side)  df$FUP_time = as.numeric(df$FUP_time)  df$rhoa_grade = factor(df$rhoa_grade)  df$aa = as.numeric(df$aa)  df$lcea = as.numeric(df$lcea)  df$scoring_approach = factor(df$scoring_approach, levels=c("KL", "Croft"))  df$location = factor(df$location, levels=c("Europe", "USA"))  df$cohort_type = factor(df$cohort_type, levels=c("Open", "Closed"))  # Create stratified cross validation split  df <- fold(data = df, num_col = 'rhoa_risk', id_col = 'person_id', k = 5)  SCV_Train1 = subset(df, df$fold != 1) # Dataset for model building  SCV_Test1 = subset(df, df$fold == 1) # Dataset for model testing  # Fit models with glmer function of lme4 on training dataset SCV_Train1  GLMM1 <- glmer(rhoa_risk ~ age + bmi + sex + hip_side + FUP_time   + (1 \| cohort_name/person_id),  family = binomial, nAGQ = 1, control = glmerControl(optimizer='nlminbwrap', optCtrl=list(maxfun=1000000)), data = SCV_Train1)  GLMM2 <- glmer(rhoa_risk ~ age + bmi + sex + hip_side + FUP_time + rhoa_grade  + (1 \| cohort_name/person_id),  family = binomial, nAGQ = 1, control = glmerControl(optimizer='nlminbwrap', optCtrl=list(maxfun=1000000)), data = SCV_Train1)  GLMM3 <- glmer(rhoa_risk ~ age + bmi + sex + hip_side + FUP_time + rhoa_grade + aa  + ns(lcea, df=2) + (1 \| cohort_name/person_id),  family = binomial, nAGQ = 1, control = glmerControl(optimizer='nlminbwrap', optCtrl=list(maxfun=1000000)), data = SCV_Train1)  GLMM4 <- glmer(rhoa_risk ~ age + bmi + sex + hip_side + FUP_time + rhoa_grade + aa  + ns(lcea, df=2) + cohort_type + location + scoring_approach  + (1 \| cohort_name/person_id),  family = binomial, nAGQ = 1, control = glmerControl(optimizer='nlminbwrap', optCtrl=list(maxfun=1000000)), data = SCV_Train1)  # Predict probability on SCV_Train1 with GLMM1  pred_proba_cohort_specific_train <- predict(GLMM1, SCV_Train1, type="response", allow.new.levels=TRUE)  pred_proba_marginal_train <- predict(GLMM1, SCV_Train1, type="response", allow.new.levels=TRUE, re.form=NA)  # Predict probability on SCV_Test1 with GLMM1  pred_proba_cohort_specific_test <- predict(GLMM1, SCV_Test1, type="response", allow.new.levels=TRUE)  pred_proba_marginal_test <- predict(GLMM1, SCV_Test1, type="response",  allow.new.levels=TRUE, re.form=NA) |
| --- |

## Leave-one-cohort-out cross validation

Table S3 - Descriptive statistics on datasets used for model building and testing


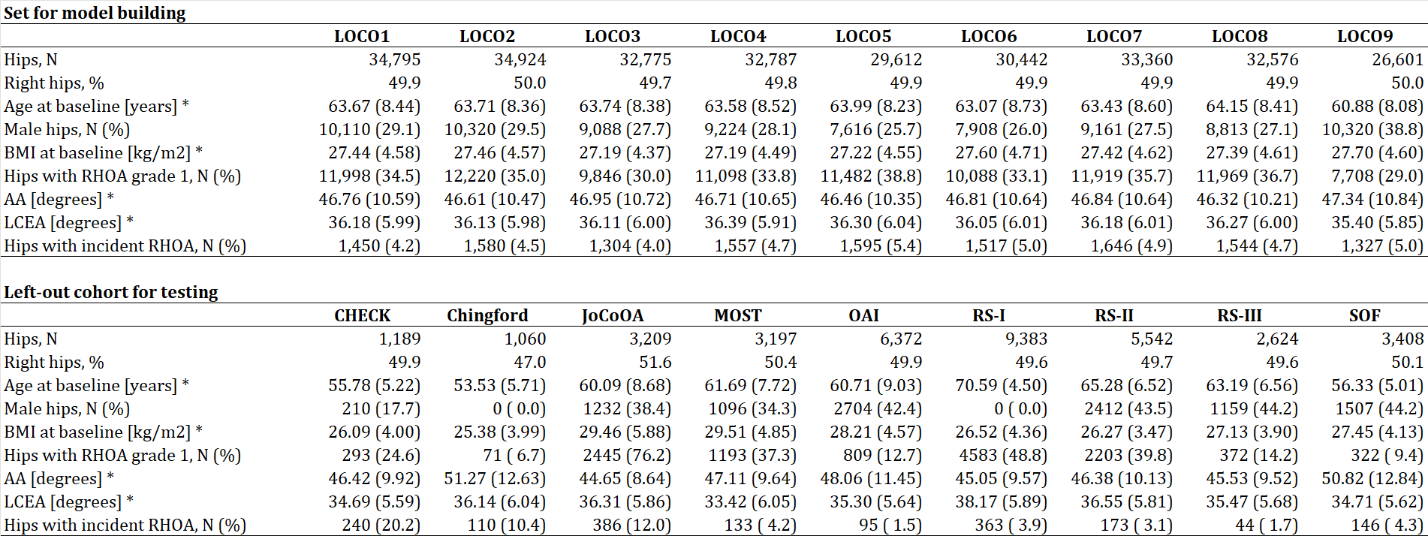


*Cohort Hip and Cohort Knee (CHECK), Chingford Study (Chingford), Johnston County Project (JoCoOA), Multi-center Osteoarthritis Study (MOST), OsteoArthritis Initiative (OAI), Rotterdam Study-I (RS-I), Rotterdam Study-II (RS-II), Rotterdam Study-III (RS-III), Study of Osteoporotic Fractures (SOF), body mass index (BMI), radiographic hip osteoarthritis (RHOA), alpha angle (AA), lateral center edge angle (LCEA)*

Table S4 – Beta values of each of the considered risk-factors within the GLMM during leave-one-cohort-out cross-validation. Continuous parameters are standardized with given mean (SD) values


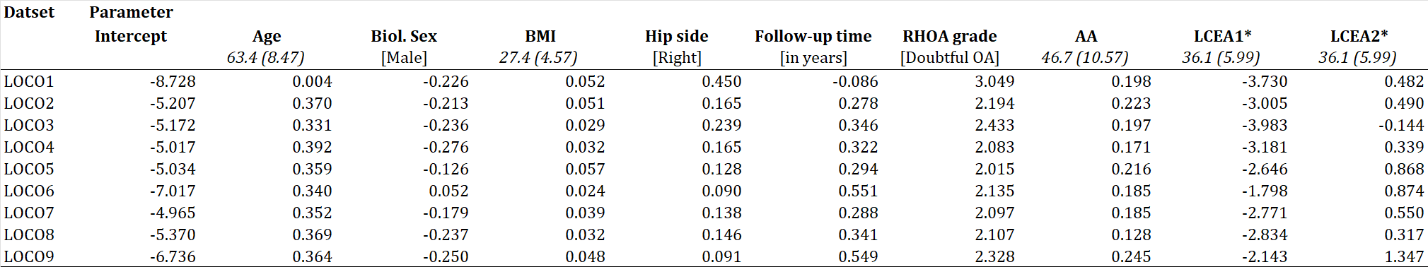


*Leave-one-cohort-out (LOCO), body mass index (BMI), radiographic hip osteoarthritis (RHOA), alpha angle (AA), lateral center edge angle (LCEA)*

** variable is modelled with natural cubic splines (df = 2), first and second beta values are given*

CodeBlock S2 – R script for building model GLMM3 and predicting the risk

| # Load all packages  library(readxl)  library(lme4)  library(dplyr)  library("splines")  library(groupdata2)  set.seed(1)  # Import dataset for analysis & select complete cases  df = read_excel("PATH TO DATASET")  df <- df[complete.cases(df), ]  # Transform data into right format  df$cohort_name = factor(df$cohort_name,  levels=c("CHECK","CHINGFORD","JOCO","MOST","OAI","SOF","RS1", "RS2","RS3"))  df$person_id = factor(df$person_id)  df$age = as.numeric(df$age)  df$bmi = as.numeric(df$bmi)  df$sex <- factor(df$sex)  df$hip_side = factor(df$hip_side)  df$FUP_time = as.numeric(df$FUP_time)  df$rhoa_grade = factor(df$rhoa_grade)  df$aa = as.numeric(df$aa)  df$lcea = as.numeric(df$lcea)  df$scoring_approach = factor(df$scoring_approach, levels=c("KL", "Croft"))  df$location = factor(df$location, levels=c("Europe", "USA"))  df$cohort_type = factor(df$cohort_type, levels=c("Open", "Closed"))  # Create leave-one-cohort-out cross validation split  LOCO1 = subset(df, df$cohort_name != "CHECK") # Dataset for model building  CHECK = subset(df, df$cohort_name == "CHECK") # Dataset for model testing  # Fit models with glmer function of lme4 on training dataset LOCO1  GLMM3 <- glmer(rhoa_risk ~ age + bmi + sex + hip_side + FUP_time + rhoa_grade + aa + ns(lcea, df=2)  + (1 \| cohort_name/person_id),  family = binomial, nAGQ = 1, control = glmerControl(optimizer='nlminbwrap', optCtrl=list(maxfun=1000000)), data = LOCO1)  # Predict on LOCO1 with GLMM3  pred_proba_cohort_specific_train <- predict(GLMM3, LOCO1, type="response",  allow.new.levels=TRUE)  pred_proba_marginal_train <- predict(GLMM3, LOCO1 , type="response",  allow.new.levels=TRUE, re.form=NA))  # Predict on CHECK with GLMM3  pred_proba_cohort_specific_test <- predict(GLMM3, CHECK, type="response",  allow.new.levels=TRUE)  pred_proba_marginal_test <- predict(GLMM3, CHECK, type="response",  allow.new.levels=TRUE, re.form=NA) |
| --- |

*CodeBlock S3 – Python script for building Random Forest model and predicting the risk*

| import pandas as pd  import numpy as np  from sklearn.ensemble import RandomForestClassifier  df = pd.read_excel(“PATH TO DATASET”)  df = df.dropna(axis = 0, how = 'any')  df = pd.get_dummies(df, columns=['cohort_name', 'hip_side', 'sex']) #one-hot encode  # Define train and test dataframes with LOCO1 and CHECK dataframes  LOCO1 = df[df['cohort_name_CHECK'] != 1]  CHECK = df[df['cohort_name_CHECK'] == 1]  # select factors of interest  exp_set = ['age', 'bmi', 'hip_side_right', 'sex_Male', 'FUP_time', 'rhoa_grade', 'aa', 'lcea', 'cohort_name_CHECK', 'cohort_name_CHINGFORD', 'cohort_name_JOCO', 'cohort_name_MOST', 'cohort_name_OAI', 'cohort_name_RS1', 'cohort_name_RS2', 'cohort_name_RS3', 'cohort_name_SOF']  X_train = LOCO1[exp_set]  y_train = np.array(LOCO1 ['rhoa_risk'])  X_test = CHECK[exp_set]  y_test = np.array(CHECK['rhoa_risk'])  # Fit model with RandomForestClassifier function of sklearn  rfc = RandomForestClassifier(random_state=42, max_depth = 10, n_estimators=200, bootstrap=True, max_features='sqrt', min_samples_leaf=8)  rfc.fit(X_train, y_train)  # Predict probabilities for the train set  pred_proba_train = rfc.predict_proba(X_train)[:,1]  # Predict probabilities for the test set  pred_proba_test = rfc.predict_proba(X_test)[:,1] |
| --- |


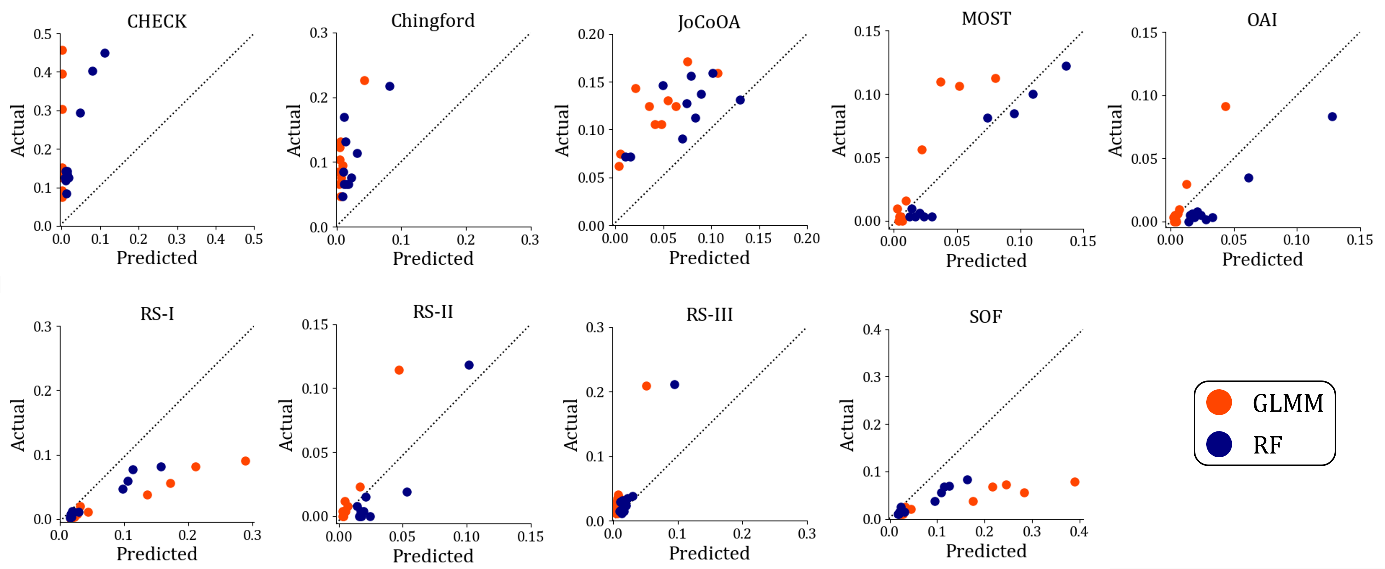


Figure S1 - Calibration plots for both the GLMM (orange) and RF (blue) based risk prediction models for each left-out cohort in leave-one-out cross validation. Each dot describes the mean predicted probability versus the actual prevalence in 10% of all hips in that cohort (ordered by predicted probability).

Cohort Hip and Cohort Knee (CHECK), Chingford Study (Chingford), Johnston County Project (JoCoOA), Multi-center Osteoarthritis Study (MOST), OsteoArthritis Initiative (OAI), Rotterdam Study-I (RS-I), Rotterdam Study-II (RS-II), Rotterdam Study-III (RS-III), Study of Osteoporotic Fractures (SOF), generalized linear mixed model (GLMM), random forest (RF)


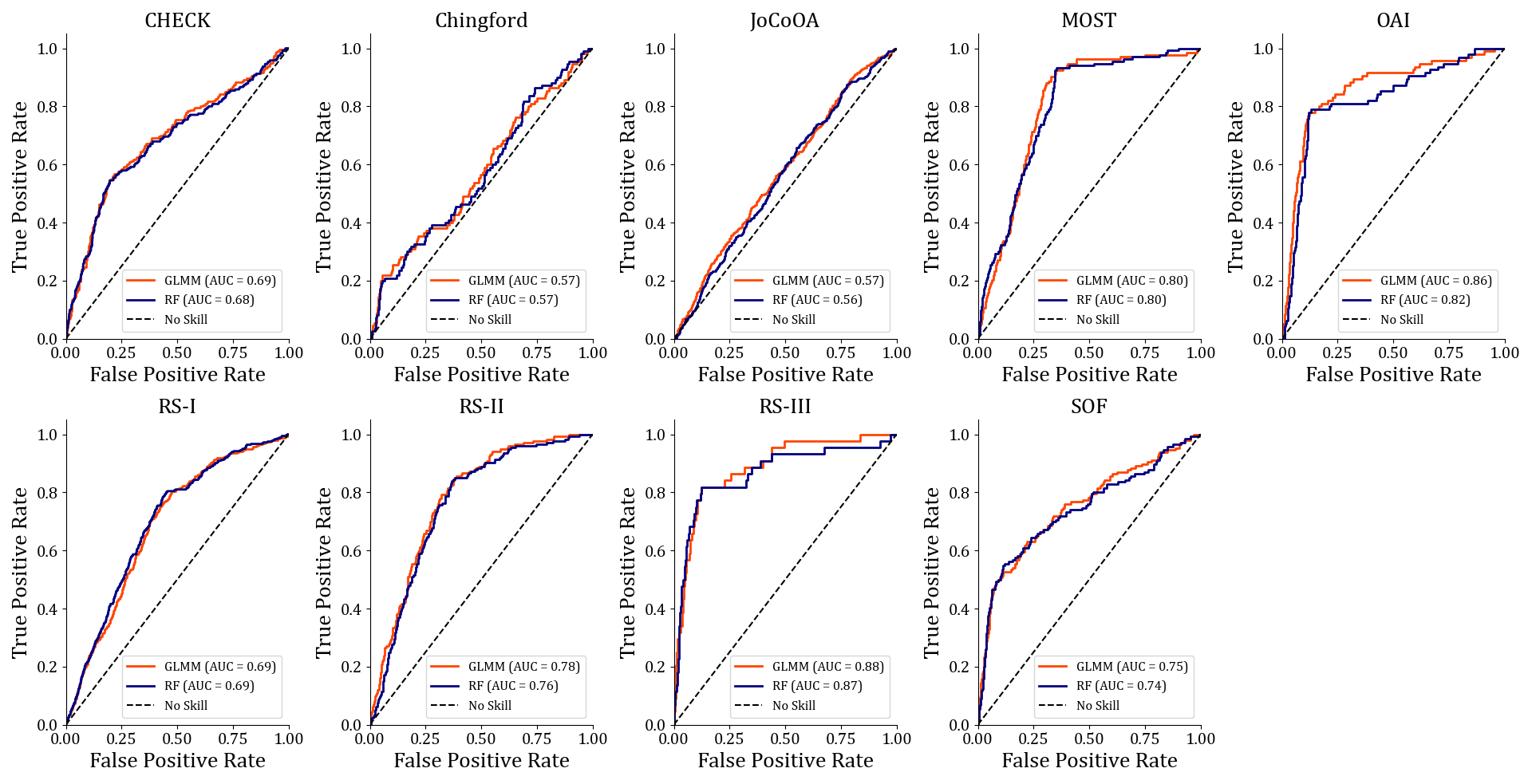


Figure S2 - ROC curves of the GLMMs (orange) and RF models (blue) per left-out cohort

*Cohort Hip and Cohort Knee (CHECK), Chingford Study (Chingford), Johnston County Project (JoCoOA), Multi-center Osteoarthritis Study (MOST), OsteoArthritis Initiative (OAI), Rotterdam Study-I (RS-I), Rotterdam Study-II (RS-II), Rotterdam Study-III (RS-III), Study of Osteoporotic Fractures (SOF), generalized linear mixed model (GLMM), random forest (RF)*

## Distribution of hip morphology measurements


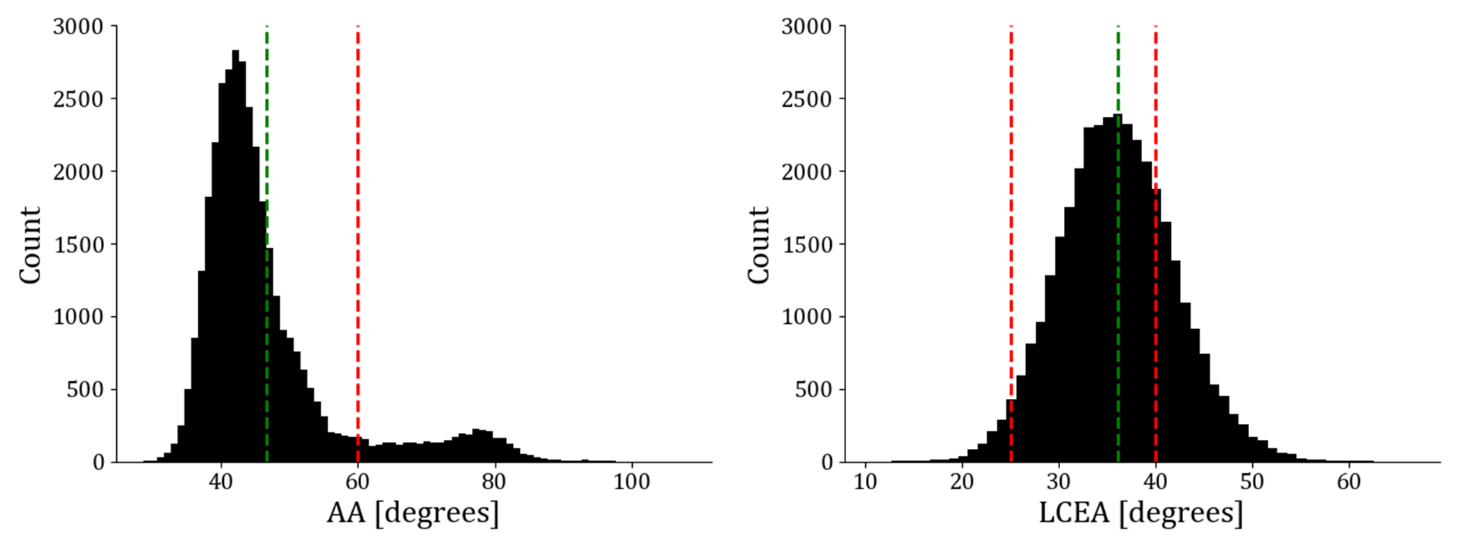


Figure S3 – Histograms of the data distribution of the alpha angle (AA, left) and the lateral center edge angle (LCEA, right) for the included dataset. The green dotted lines indicate the mean values of the population. The red dotted lines indicate clinically relevant thresholds for cam morphology (AA ≥ 60°), acetabular dysplasia (LCEA ≤ 25°), and pincer morphology (LCEA≥40°)
